# Dynamin Binding Protein Is Required for *Xenopus laevis* Kidney Development

**DOI:** 10.3389/fphys.2019.00143

**Published:** 2019-02-26

**Authors:** Bridget D. DeLay, Tanya A. Baldwin, Rachel K. Miller

**Affiliations:** ^1^Department of Pediatrics, McGovern Medical School, Pediatric Research Center, University of Texas Health Science Center, Houston, TX, United States; ^2^Department of Integrative Biology and Pharmacology, McGovern Medical School, University of Texas Health Science Center, Houston, TX, United States; ^3^Program in Biochemistry and Cell Biology, Graduate School of Biomedical Sciences, University of Texas MD Anderson Cancer Center, University of Texas Health Science Center, Houston, TX, United States; ^4^Program in Genetics and Epigenetics, Graduate School of Biomedical Sciences, University of Texas MD Anderson Cancer Center, University of Texas Health Science Center, Houston, TX, United States; ^5^Department of Genetics, University of Texas MD Anderson Cancer Center, Houston, TX, United States

**Keywords:** Dnmbp, Tuba, *Xenopus*, nephrogenesis, pronephros, CRISPR

## Abstract

The adult human kidney contains over one million nephrons, with each nephron consisting of a tube containing segments that have specialized functions in nutrient and water absorption and waste excretion. The embryonic kidney of *Xenopus laevis* consists of a single functional nephron composed of regions that are analogous to those found in the human nephron, making it a simple model for the study of nephrogenesis. The exocyst complex, which traffics proteins to the cell membrane in vesicles via CDC42, is essential for normal kidney development. Here, we show that the CDC42-GEF, dynamin binding protein (Dnmbp/Tuba), is essential for nephrogenesis in *Xenopus*. *dnmbp* is expressed in *Xenopus* embryo kidneys during development, and knockdown of Dnmbp using two separate morpholino antisense oligonucleotides results in reduced expression of late pronephric markers, whereas the expression of early markers of nephrogenesis remains unchanged. A greater reduction in expression of markers of differentiated distal and connecting tubules was seen in comparison to proximal tubule markers, indicating that Dnmbp reduction may have a greater impact on distal and connecting tubule differentiation. Additionally, Dnmbp reduction results in glomus and ciliary defects. *dnmbp* knockout using CRISPR results in a similar reduction of late markers of pronephric tubulogenesis and also results in edema formation in later stage embryos. Overexpression of *dnmbp* in the kidney also resulted in disrupted pronephric tubules, suggesting that *dnmbp* levels in the developing kidney are tightly regulated, with either increased or decreased levels leading to developmental defects. Together, these data suggest that Dnmbp is required for nephrogenesis.

## Introduction

Kidney development is conserved in amphibians and mammals, making *Xenopus* embryos a good model for studying nephrogenesis. Mammalian kidney development proceeds through three stages: the pronephros, mesonephros, and metanephros (Vize et al., 1997). Similarly, amphibian embryos have a pronephros, and adults have a metanephros (Vize et al., 1995, 1997). The basic unit of filtration for all kidney forms is the nephron, with the same signaling cascades and inductive events leading to nephrogenesis in mammals and amphibians (Brandli, 1999; Hensey et al., 2002). The *Xenopus* pronephros consists of a single, large, functional nephron (Brennan et al., 1998; Carroll et al., 1999), making it a simple model for studying vertebrate nephron development. Additionally, the *Xenopus* tadpole epidermis is transparent and the kidney is located just under the epidermis, allowing visualization of the pronephros without dissection (Carroll et al., 1999). It is also possible to easily modulate gene expression in *Xenopus* embryos through overexpression, knockdown and knockout experiments via microinjection of RNA constructs, antisense morpholino oligonucleotides (MOs) and CRISPR constructs (Miller et al., 2011; Corkins et al., 2018; DeLay et al., 2018b). The established cell fate maps of the early *Xenopus* embryo facilitate tissue-targeted modulation of gene expression by microinjection into the appropriate blastomere (Moody, 1987a,b; DeLay et al., 2016, 2018b). Taken together, *Xenopus* is a powerful model for studying essential nephrogenesis genes.

One gene that plays an essential role in kidney development is *cdc42*. Cdc42 is a Rho family small GTPase that was first discovered in *Saccharomyces cerevisiae* (Johnson and Pringle, 1990). It plays a role in cell migration, polarity, differentiation and proliferation, as well as branching of blood vessels and regulation of actin dynamics (Melendez et al., 2013; Schulz et al., 2015; Mizukawa et al., 2017; Nguyen et al., 2017; Lavina et al., 2018). Cdc42 is a molecular switch that cycles between active (GTP-bound) and inactive (GDP-bound) states through its interaction with guanine exchange factors (GEFs) and GTPase activating proteins (GAPs) (Bishop and Hall, 2000; Schmidt and Hall, 2002). While GAPs increase the intrinsic GTPase activity of CDC42, GEFs exchange GDP bound to Cdc42 for GTP and assemble complexes between Cdc42, scaffold proteins and kinases (Cerione, 2004).

Loss of Cdc42 in the mouse ureteric bud leads to abnormal nephron tubulogenesis due to branching, polarity and cytoskeletal defects, while loss of Cdc42 in the mouse metanephric mesenchyme results in failure of the renal vesicle and S-shaped body to develop (Elias et al., 2015). Similarly, loss of Cdc42 in the distal tubules of mouse kidney leads to death within a few weeks of birth due to kidney failure, cyst development and a decrease in ciliogenesis within the kidney cysts (Choi et al., 2013). Knockdown of Cdc42 via MO in zebrafish leads to dilated kidney tubules, glomerulus defects and disorganized cilia within kidney tubules (Choi et al., 2013).

Although Cdc42 localizes on the apical surface of the kidney tubule epithelium, it needs to be activated by a GEF in order to regulate tubulogenesis and ciliogenesis (Martin-Belmonte et al., 2007; Zuo et al., 2011). Dynamin binding protein (Dnmbp, Tuba) is a Cdc42-specific GEF that is known to be concentrated on the apical surface of kidney epithelial cells (Otani et al., 2006; Qin et al., 2010). Knockdown of Dnmbp in MDCK cells leads to a decrease in cilia, polarity defects and inhibition of tubulogenesis, similar to that seen when Cdc42 is knocked down (Zuo et al., 2011; Baek et al., 2016). Here, we demonstrate that knockdown, CRISPR knockout and overexpression of *dnmbp* lead to tubulogenesis and cilia defects in *Xenopus* pronephric kidneys, indicating that this protein is required for nephrogenesis.

## Materials and Methods

### Embryos

Adult pigmented *X. laevis* were purchased from Nasco (LM00531MX). Eggs were obtained from female frogs, fertilized *in vitro* and the embryos reared as described previously (Sive et al., 2000). The Center for Laboratory Animal Medicine Animal Welfare Committee at the University of Texas Health Science Center at Houston, which serves as the Institutional Animal Care and Use Committee, approved this protocol (protocol #AWC-16-0111).

### Western Blots

Embryos were collected at various stages (Nieuwkoop and Faber, 1994) for lysate creation. Protein lysates from 20 pooled embryos of the same stage were created as described previously (Kim et al., 2002), and one embryo equivalent was added per lane of an 8% SDS-PAGE polyacrylamide gel. Following transblotting of the protein onto a 0.45 μm PVDF membrane (Thermo Scientific), the blot was blocked for 3 h in KPL block (SeraCare) at room temperature. After blocking, the membrane was incubated overnight at 4°C in 1:500 mouse anti-Dnmbp antibody (Abcam 88534) or 1:1000 rabbit anti-GAPDH antibody (Santa Cruz FL-335). Blots were rinsed with TBST and incubated in 1:5000 goat anti-mouse or goat anti-rabbit IgG horseradish peroxidase secondary antibody (BioRad, Hercules, CA) for 2 h at room temperature. Blots were rinsed again in TBST and imaged using enhanced chemiluminescence (Pierce Supersignal West Pico) on a BioRad ChemiDoc XRS+.

### *In situ* Hybridization

A DIG RNA labeling kit (Roche) was used to generate digoxigenin-labeled RNA probes for *in situ* hybridization. Constructs were linearized prior to generating probes using the listed enzyme and polymerase: *atp1a1*-antisense *Sma*I/T7 (Eid and Brandli, 2001), *lhx1*-antisense *Xho*I/T7 (Taira et al., 1992; Carroll and Vize, 1999), *hnf1β*-antisense *Sma*I/T7 (Demartis et al., 1994), *nphs1*-antisense *Sma*I/T7 (Gerth et al., 2005), *clcnkb*-antisense *Eco*RI/T7 (Vize, 2003), *slc5a1*-antisense *Sma*I/T7 (Zhou and Vize, 2004), and *pax2*-antisense *Eco*RI/T7 (Carroll and Vize, 1999).

Digoxigenin-labeled *dnmbp* RNA probes were generated by first extracting DNA from stage 40 embryos as previously described (Bhattacharya et al., 2015). Regions of *dnmbp.L* and *dnmbp.S* were amplified from embryo DNA by PCR using the following primers: *dnmbp.L*-sense-Sp6 (5′-CTAGCATTTAGGTGACACTATAGGTCAAAGGACACTCGAAACAC-3′), *dnmbp.L*-antisense-T7 (5′-CTAGCTAATACGACTCACTATAGAGAAACATTCGTCTCGCGAGG-3′), *dnmbp.S*-sense-Sp6 (5′-CTAGCATTTAGGTGACACTATAGGTTAAAGGACACTCGAAACAC-3′) and *dnmbp.S*-antisense-T7 (5′-CTAGCTAATACGACTCACTATAGAGAAACGTTCGTGGAGGGTAC-3′). PCR products were transcribed to create digoxigenin-labeled RNA probes using a DIG RNA labeling kit (Roche) and the appropriate polymerase (T7 or Sp6).

### MOs and RNA Constructs

Two translation-blocking MOs were designed to target the 5′ untranslated region of *dnmbp*: Dnmbp MO 1,5′-TCGAACCACCGATCCCACCTCCATC-3′; Dnmbp MO 2,5′-ACCACCGACCCCACCTCCATCCTAA-3′. A Standard control MO (5′-CCTCTTACCTCAGTTACAATTTATA-3′) was used as a control for all MO experiments. MOs were ordered from Genetools. Single cell embryos were injected with 40 ng MO for Western blot analysis and 8-cell embryos were injected with 20 ng MO for phenotypic analysis.

Human *DNMBP* RNA was created by linearizing pcDNA3-HA-Tuba (Addgene plasmid 22214) DNA with *Xba*I (Salazar et al., 2003). Capped RNA for rescue and overexpression experiments was transcribed and purified from the linearized DNA using a T7 mMachine mMessage kit (Ambion). A pCS2-*β-galactosidase* construct was obtained from the McCrea laboratory for use as a control for rescue and overexpression experiments (Lyons et al., 2009; Miller et al., 2011). *β-galactosidase* RNA was transcribed from plasmid DNA linearized with *Not*I using a Sp6 mMachine mMessage kit (Ambion).

### sgRNA Design and Creation

One sgRNA that was complimentary against both homeologs of *dnmbp* (5′-CTAGCTAATACGACTCACTATAGGGAGCGCTCCTGGTTCATGGGGTTTTAGAGCTAGAAATAGCAAG-3′) was designed as previously reported (DeLay et al., 2018b). A sgRNA against *slc45a2* was generated for use as a control (DeLay et al., 2018b). DNA templates for sgRNAs were produced by PCR, and T7 polymerase was used to transcribe sgRNA from the DNA templates as previously described (Bhattacharya et al., 2015; DeLay et al., 2018b). For long-term storage, sgRNA was diluted to 1000 ng/μL and kept at -80°C. For working stocks, sgRNA was diluted to 500 ng/μL and stored in 5 μL aliquots of at -20°C. Working stock aliquots were limited to five freeze-thaw cycles prior to disposal. Single cell and 8-cell embryos were injected with 1 ng Cas9 protein and 500 pg sgRNA.

### CRISPR Genomic Analysis

Embryos injected at the 1-cell stage with 1 ng Cas9 protein and 500 pg sgRNA were reared to stage 40. DNA was extracted from individual embryos as previously described (Bhattacharya et al., 2015), and the region surrounding the sgRNA binding site was amplified by PCR as previously described (DeLay et al., 2018b). *dnmbp.L* DNA was amplified using nested PCR. The outer set of primers used were *dnmbp.L-*outer*-*forward (5′-AGCTGACCCCATCTTAAAACAA-3′) and *dnmbp.L*-reverse (5′-GTTTTTAGCTGCTTGGCTCAGT-3′). Following the outer PCR reaction, the resulting PCR product was used to amplify *dnmbp.L* using primers *dnmbp.L*-reverse and *dnmbp.L-*inner-forward (5′-TTCATGGCCTCTCCTACTCATT-3′). *dnmbp.L* was sequenced using primer *dnmbp.L-*outer-forward. *dnmbp.*S DNA was amplified with primers *dnmbp.S*-forward (5′-GACCCCATAATTGAGCCATAAG-3′) and *dnmbp.S-*reverse (5′-CAGTGGTTTTGACGATTGTAGC-3′) and sequenced using *dnmbp.S-*forward. TIDE was used to determine insertion and deletion frequencies in the amplified gene region (Brinkman et al., 2014).

### Microinjection

Individual blastomeres were microinjected with 10 nL of injection mix as described previously (DeLay et al., 2016). Blastomere V2 of 8-cell embryos was injected to target the kidney (Moody, 1987a). Cas9 protein (CP01; PNA Bio) and sgRNA were incubated together at room temperature for at least 5 min prior to microinjection (DeLay et al., 2018b). MOs, RNA constructs and Cas9/sgRNAs were co-injected with either membrane-RFP RNA, Alexa Fluor 488 fluorescent dextran or rhodamine dextran as a tracer (Davidson et al., 2006; DeLay et al., 2016, 2018b). For edema experiments, embryos were injected into both ventral blastomeres at the 4-cell stage.

### Immunostaining

Staged embryos (Nieuwkoop and Faber, 1994) were fixed (DeLay et al., 2016) prior to immunostaining as described previously (Lyons et al., 2009). The lumens of the proximal kidney tubules were labeled using 3G8 antibody (1:30) and the distal and connecting kidney tubules were labeled using 4A6 antibody (1:5) (Vize et al., 1995). Additionally, proximal tubules were detected using fluorescein-labeled *Erythrina cristagalli* lectin (50 μg/mL; Vector Labs). Somites were labeled using antibody 12/101 (1:100) (Kintner and Brockes, 1984) and membrane-RFP tracer was labeled with anti-RFP antibody (1:250; MBL International PM005). Kidney, somite and membrane-RFP tracer staining were visualized using goat anti-mouse IgG Alexa 488 (1:2000; Invitrogen) and goat anti-rabbit IgG Alexa 488 and Alexa 555 (1:2000; Invitrogen).

### Imaging

Embryo phenotypes were scored and *in situ* images were taken using an Olympus SZX16 fluorescent stereomicroscope with an Olympus DP71 camera or a Leica S8 A80 stereomicroscope with a Leica MC120 HD camera. Confocal images were taken with a Zeiss LSM800 microscope. Adobe Photoshop and Illustrator CS6 were used to process images and create figures.

## Results

### Dnmbp Is Expressed in the Developing *Xenopus* Pronephros

**FIGURE 1:**
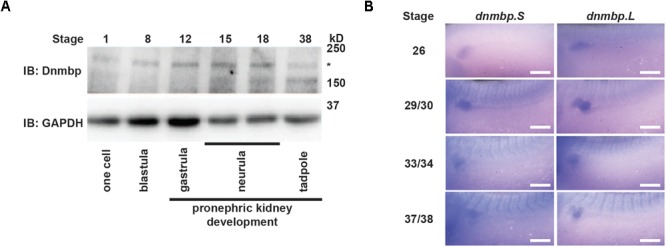
Dnmbp is present throughout *Xenopus* pronephric development. **(A)** Western blot showing that Dnmbp is present in embryos ranging from stage one to stage 38. ^∗^ indicates Dnmbp. **(B)** *In situ* hybridization using probes against *dnmbp.S* and *dnmbp.L* showing *dnmbp* expression in the developing pronephros from stage 26 through stage 37/38. White bar is 500 μm.

To assess whether Dnmbp protein is expressed during kidney development, protein lysates were collected from embryos at different developmental stages. Using a commercial antibody against Dnmbp, we found that Dnmbp protein (170 kD) is present in *Xenopus* embryos from single cell through tadpole stages by Western blot (Figure 1A). Importantly, Dnmbp protein was present from gastrula (stage 12) through tadpole (stage 38) stages when pronephric kidney specification and development occur.

To determine if *dnmbp* is present in the *Xenopus* kidney throughout pronephric development, embryos ranging from stage 26 to 38 were subjected to *in situ* hybridization (Figure 1B and Supplementary Figure S1). Antisense probes were created against each homeolog of *dnmbp*, and sense probes against each homeolog were used to verify that staining was specific for *dnmbp*. Starting at stage 26, both antisense probes against *dnmbp* stained the kidney tubules, with the strongest staining in the proximal tubules (Figure 1B). In addition to the kidney, the antisense *dnmbp* probes stained head structures and somites (Supplementary Figure S1). In comparison, the sense control *dnmbp* probes did not label any embryonic structures when processed in parallel to the antisense probes, indicating that the antisense *dnmbp* probe staining was specific for *dnmbp* (Supplementary Figure S1). Taken together, this indicates that *dnmbp* transcripts are present in the kidney during nephrogenesis.

### Knockdown of Dnmbp Leads to Altered Pronephric Development in *Xenopus*

**FIGURE 2:**
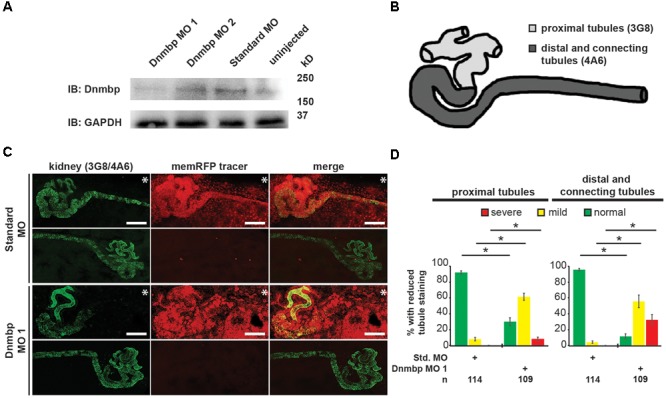
Knockdown of Dnmbp results in reduced kidney tubulogenesis. **(A)** Western blot showing the efficiency of two different MOs targeting Dnmbp. Single cell embryos were injected with 40ng of Dnmbp MO 1, Dnmbp MO 2 or Standard MO. One embryo equivalent per lane was loaded on to the SDS-PAGE gel. **(B)** Schematic of the *Xenopus* pronephros showing the proximal, distal and connecting tubule regions. **(C)** Unilateral injection of 20ng Dnmbp MO 1 into blastomere V2 at the 8-cell stage leads to defects in kidney tubulogenesis in comparison to embryos injected with Standard MO. Antibody 3G8 used to label the lumen of the proximal tubule, antibody 4A6 used to label the distal and connecting tubules. memRFP used as an injection tracer. White bar indicates 200 μm.^∗^ indicates injected side of embryo. **(D)** Knockdown of Dnmbp leads to reduced expression of differentiated kidney tubule markers in comparison to embryos injected with Standard MO. *n* = number of embryos across 3 replications. Error bars represent Standard error. ^∗^Significantly different from control, *p* < 0.05.

To determine whether Dnmbp is necessary for the development of the *Xenopus* pronephros, we examined the expression pattern of markers of differentiated kidney tubules upon depletion of Dnmbp. Dnmbp was knocked down using two different translation-blocking MOs: Dnmbp MO 1 and Dnmbp MO 2. Both MOs were designed to target the 5′ untranslated region of *dnmbp*. Knockdown in single cell embryos was confirmed by Western blot (Figure 2A) of stage 10–12 embryos in comparison to embryos injected with a Standard MO control. Dnmbp MO 1 showed a marked decrease in Dnmbp protein levels in comparison to both Standard MO and uninjected controls. Dnmbp MO 2 showed a decrease in Dnmbp in comparison to Standard MO controls, but did not show a clear reduction in comparison to uninjected controls. For this reason, phenotypic analysis was carried out for Dnmbp MO 1 knockdown embryos.

Pronephric tubule development was assessed upon Dnmbp knockdown using 3G8 and 4A6 antibodies (Vize et al., 1995), which label the differentiated proximal tubules versus the distal and connecting tubules, respectively (Figure 2B). Knockdown phenotypes of embryos injected in the left V2 blastomere at the 8-cell stage were assessed using a previously described scoring system by comparing the tubules on the MO-injected side of the embryo to the tubules on the uninjected side (DeLay et al., 2018b). Phenotypes were scored as “normal” if there was no difference between the injected and uninjected side, “mild” if there was a reduction in tubule development and/or antibody staining on the injected side in comparison to the injected side or “severe” if there was little to no tubule and/or antibody staining on the injected side of the embryo.

Knockdown of Dnmbp resulted in disrupted proximal tubule development in stage 40–41 embryos that had been injected with either Dnmbp MO in comparison to Standard MO-injected controls (Figure 2C,D and Supplementary Figure S2). The proximal tubules in Dnmbp knockdown embryos had shorter branches and were less convoluted than Standard MO-injected control embryos. Similarly, distal and connecting tubule development was disrupted upon Dnmbp knockdown, resulting in decreased convolution of the distal and connecting tubules of Dnmbp knockdown embryos in comparison with those of Standard MO-injected controls (Figure 2C,D and Supplementary Figure S2). Interestingly, there was a decrease in 4A6 staining of the distal and connecting tubules even though these tubules could be visualized using the co-injected memRFP tracer (Figure 2C and Supplementary Figure S2) indicating that the distal and connecting tubules are either absent or they are less differentiated than the tubules in the Standard MO-injected control embryos.

**FIGURE 3:**
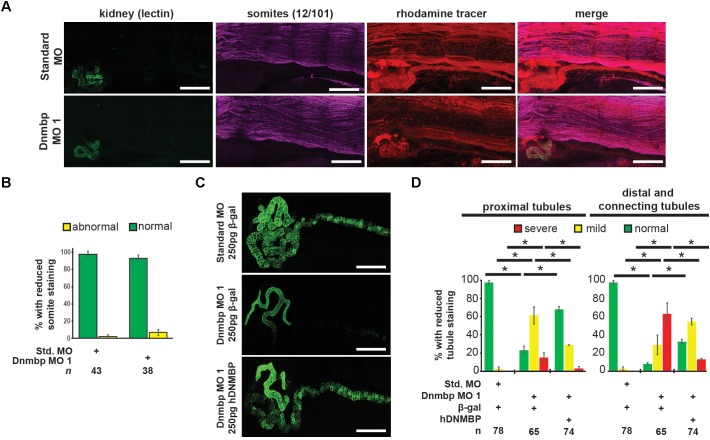
Dnmbp knockdown is specific and does not cause somite development defects. **(A)** Unilateral injection of 20 ng Dnmbp MO 1 into blastomere V2 at the 8-cell stage does not cause somite defects in comparison to embryos injected with Standard MO. 12/101 antibody labels somites, lectin labels the proximal tubule lumen, rhodamine used as a tracer. Images are stitched from 6 tiles. White bar is 200 μm. **(B)** Knockdown of Dnmbp does not lead to reduced somite development compared to embryos injected with Standard MO. **(C)** Representative embryos showing that co-injection of *β-galactosidase* RNA with Dnmbp MO 1 does leads to kidney defects in comparison to control embryos injected with Standard MO and *β-galactosidase* RNA. Co-injection of human *DNMBP* mRNA with Dnmbp MO 1 rescues the knockdown phenotype. Stage 40 embryos stained with antibody 3G8 to label the proximal tubule and antibody 4A6 to label the distal and connecting tubules. White bar is 200 μm. **(D)** Quantitation of the rescue phenotype. *n* = number of embryos across 3 replications. Error bars represent Standard error. ^∗^Significantly different, *p* < 0.05.

To assess whether the pronephric defects observed in Dnmbp knockdown embryos are secondary defects caused by defects in somite development, immunostaining was performed. Somites of stage 40–41 embryos were stained with 12/101 antibodies, and the lumen of the proximal tubules was stained with lectin. Embryos were injected with either Standard MO or Dnmbp MO 1 at the 8-cell stage (left V2 blastomere) with rhodamine used as a tracer, and somite staining on the injected side of the embryo was compared to staining on the uninjected side of the embryo. There was no difference between somite staining of Standard MO- and Dnmbp MO-injected embryos (Figure 3A,B), although lectin staining indicated that there were proximal tubule defects in the Dnmbp knockdown embryos (Figure 3A). Note that the rhodamine tracer is present in the somites as well as in the kidney, indicating that both the somites and the kidney received MO. Additionally, it is possible to visualize distal and connecting tubule defects in the Dnmbp knockdown embryos by observing the rhodamine tracer localization in the kidney. The lack of somite defects in the Dnmbp knockdown embryos indicates that the observed tubule defects are not likely to be due to somite development defects.

To assess the specificity of the Dnmbp knockdown, Dnmbp MO 1 was co-injected with human *DNMBP* RNA in an attempt to rescue the knockdown phenotype. *β-galactosidase* (*β-gal*) was used as a negative RNA control. Embryos were assessed at stages 40–41. Co-injection of Dnmbp MO 1 and *β*-*gal* RNA led to the expected decrease in proximal, distal and connecting tubule development (Figure 3C,D). Similarly, co-injection of Standard MO and β-*gal* RNA did not lead to defects in tubulogenesis (Figure 3C,D). Co-injection of Dnmbp MO 1 and human *DNMBP* RNA resulted in fewer tubulogenesis defects than in Dnmbp MO 1 and *β*-*gal* RNA control embryos, indicating that human *DNMBP* RNA is able to rescue the kidney phenotypic defects caused by the Dnmbp MO 1. This result indicates that the kidney phenotype observed is due to Dnmbp knockdown specifically.

### Knockdown of Dnmbp Leads to Disruption of Nephric Cilia Organization in *Xenopus*

**FIGURE 4:**
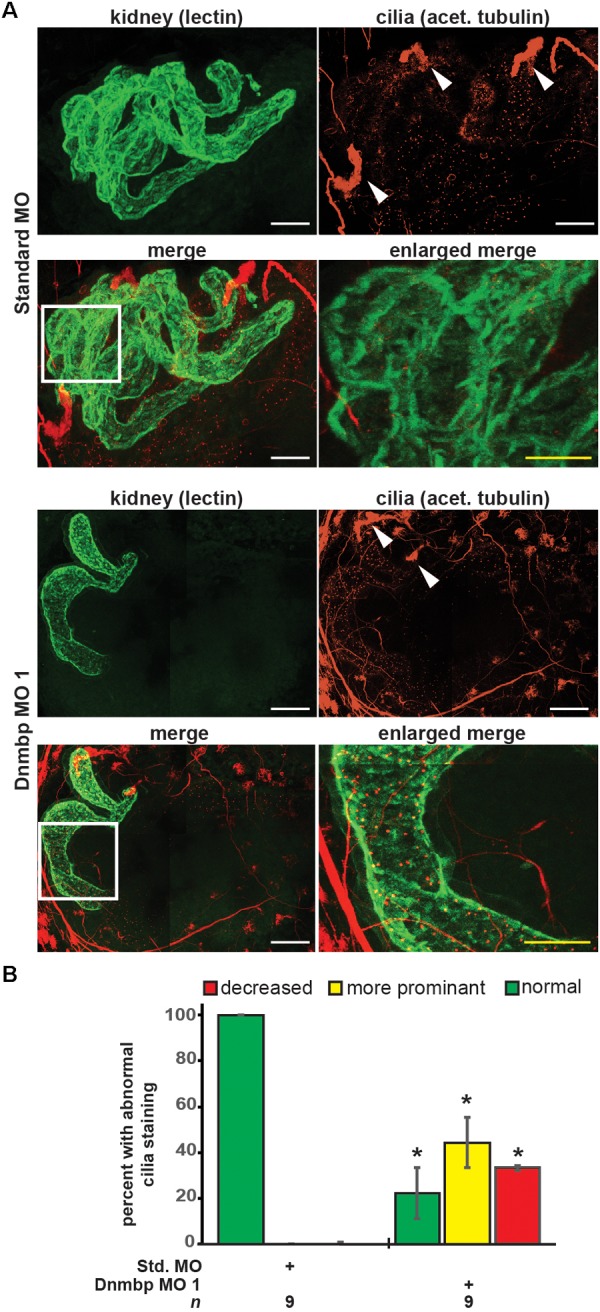
Dnmbp knockdown alters ciliogenesis. **(A)** Unilateral injection of 20ng Dnmbp MO 1 into blastomere V2 at the 8-cell stage causes altered primary ciliogenesis in comparison to embryos injected with Standard MO. Longer primary cilia are visible in the proximal tubules of the Dnmbp knockdown embryos (“more prominent cilia” phenotype). Acetylated tubulin antibody used to label cilia and nerves and lectin used to label the proximal tubule lumen. Images are stitched from 6 tiles. White bar is 50 μm, yellow bar is 25 μm. White arrowhead indicates nephrostome. White box indicates region represented in the enlarged merge panel. **(B)** Knockdown of Dnmbp leads to altered primary cilia development compared to embryos injected with Standard MO. *n* = number of embryos across 3 replications. Error bars represent Standard error. ^∗^Significantly different from control, *p* < 0.05.

As defects in cilia development have been reported in zebrafish in which Dnmbp has been knocked down (Baek et al., 2016), cilia development in the pronephros was assessed. Embryos were injected with Standard MO or Dnmbp MO 1 in the left V2 blastomere at the 8-cell stage, reared to stage 40–41 and stained with lectin to label the proximal tubules and an acetylated tubulin antibody to label cilia and nerves. Control embryos showed very short cilia in the proximal tubules and longer-appearing cilia in the distal and connecting tubules (Figure 4A). 78% (7/9) of Dnmbp knockdown embryos displayed primary cilia defects in the kidney tubules (Figure 4B). Of these, the abnormal primary cilia phenotypes were broken down into two distinct groups: “more prominent cilia” and “reduced cilia.” 44% (4/9) of Dnmbp knockdown embryos displayed more prominent cilia within the proximal tubules in comparison to the controls, while the cilia in the distal and connecting tubules appeared to be normal (Figure 4A, compare panels labeled enlarged merge). 33% (3/9) of Dnmbp knockdown embryos displayed reduced cilia in the proximal, distal and intermediate tubules in comparison to controls and 22% (2/9) of Dnmbp knockdown embryos showed no obvious primary cilia defect. Cilia were present on the nephrostomes of all control and Dnmbp knockdown embryos (Figure 4A, white arrowheads) and multiciliated cells on the epidermis of all of the Dnmbp knockdown embryos appeared to be normal (data not shown). These data suggest that there is a primary cilia defect in the kidneys of Dnmbp knockdown embryos, while motile cilia appear to be grossly unaffected.

### CRISPR *dnmbp* Knockout Phenocopies Dnmbp Knockdown

**FIGURE 5:**
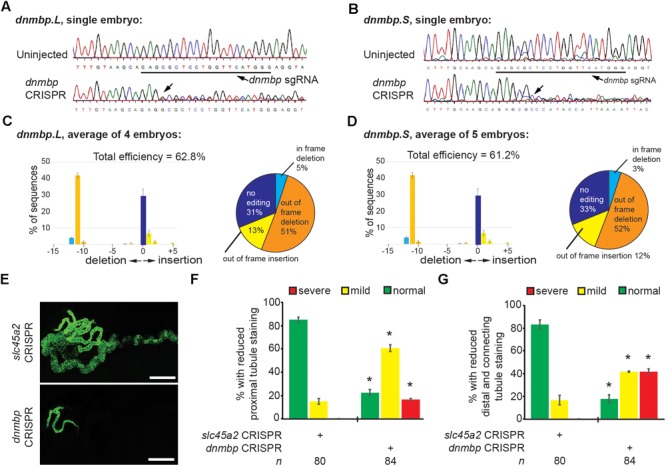
sgRNA targeting *dnmbp* efficiently edits *Xenopus* embryo DNA. Stage 40 embryos injected with *dnmbp* sgRNA and Cas9 protein at the 1-cell stage. **(A)** Chromatogram showing CRISPR editing of *dnmbp.L* in a single embryo. The underlined sequence corresponds to the *dnmbp* sgRNA binding sequence, and the arrow indicates sequence degradation due to CRISPR. **(B)** Chromatogram showing CRISPR editing of *dnmbp.S* in a single embryo. The underlined sequence corresponds to the *dnmbp* sgRNA binding sequence, and the arrow indicates sequence degradation due to CRISPR. **(C)** TIDE analysis of *dnmbp.L* sequence trace degradation after the expected Cas9 cut site. ^∗^*p* < 0.001. Percentage of *dnmbp.L* DNA sequence containing insertions and deletions. Bars indicate the mean of the percent of insertion/deletion sequences from four embryos, with the error bars representing the Standard error of the mean. Results are the mean of sequencing data from four embryos. **(D)** TIDE analysis of *dnmbp.S* sequence trace degradation after the expected Cas9 cut site. ^∗^*p* < 0.001. Percentage of *dnmbp.S* DNA sequence containing insertions and deletions. Bars indicate the mean of the percent of insertion/deletion sequences from four embryos, with the error bars representing the Standard error of the mean. Results are the mean of sequencing data from four embryos. **(E)** Representative stage 40 embryos showing that 8-cell targeted knockout of *dnmbp* leads to disrupted kidney tubulogenesis in comparison to *slc45a2* knockout controls. Antibody 3G8 labels the proximal tubule lumen and antibody 4A6 labels cell membranes of the distal and connecting tubules. White bar is 200 μm. **(F)** Knockout of *dnmbp* reduces proximal tubule development. **(G)** Knockout of *dnmbp* reduces distal and intermediate tubule development. **(F,G)** *n* = number of embryos across 3 replications. Error bars represent Standard error. ^∗^Significantly different from control, *p* < 0.05.

To further confirm that loss of *dnmbp* leads to kidney tubulogenesis defects, we designed a single sgRNA with complete complementarity to both homeologs of *dnmbp*. Embryos were injected with 500 pg *dnmbp* sgRNA and 1 ng Cas9 protein, and a region surrounding the sgRNA binding site was amplified by PCR. Different sets of sequencing primers, each specific for one of the two *dnmbp* homeologs, were used to distinguish between *dnmbp.L* and *dnmbp.S* DNA sequences (Supplementary Figure S3). Sequences were analyzed using TIDE, a web-based tool that allows the user to easily compare DNA sequence trace decomposition around the predicted Cas9 cut site to an unedited control sequence while providing output on indel composition and percent of DNA edited (Brinkman et al., 2014; DeLay et al., 2018b). TIDE analysis indicated that the *dnmbp* sgRNA efficiently knocked out both homeologs in F0 embryos (Figure 5). Individual embryo sequence traces showed an increase in sequence trace decomposition around the expected sgRNA binding site, indicating CRISPR editing (Figure 5A,B). Overall, CRISPR knockout of *dnmbp* resulted in 62.8% editing efficiency of the *dnmbp.L* homeolog and 61.2% editing efficiency of the *dnmbp.S* homeolog (Figure 5C,D). The most common mutation for both homeologs was an 11 base pair out of frame deletion (Figure 5C,D).

Embryos were injected at the 8-cell stage (left V2 blastomere) with 1 ng Cas9 protein and either 500 pg *dnmbp* sgRNA or control *slc45a2* sgRNA and reared to stage 40 to assess kidney development (DeLay et al., 2018b). *slc45a2* was knocked out as a control because loss of this gene leads to decreased pigmentation of the eyes and melanocytes, but does not alter kidney development (DeLay et al., 2018b). Therefore, knockout of *slc45a2* was used as a negative control with which to compare *dnmbp* knockout embryo phenotype.

Proximal tubule staining of *dnmbp* knockout embryos using 3G8 antibodies indicated a reduction in proximal tubule branching and convolution in comparison to *slc45a2* knockout controls (Figure 5E,F). Distal and connecting tubule development was also disrupted in *dnmbp* knockout embryos in comparison to *slc45a2* knockout controls, with a decrease in 4A6 staining indicating less differentiated distal and connecting tubules (Figure 5E,G). The phenotype observed in *dnmbp* knockout embryos was similar to that seen in MO knockdown embryos.

**FIGURE 6:**
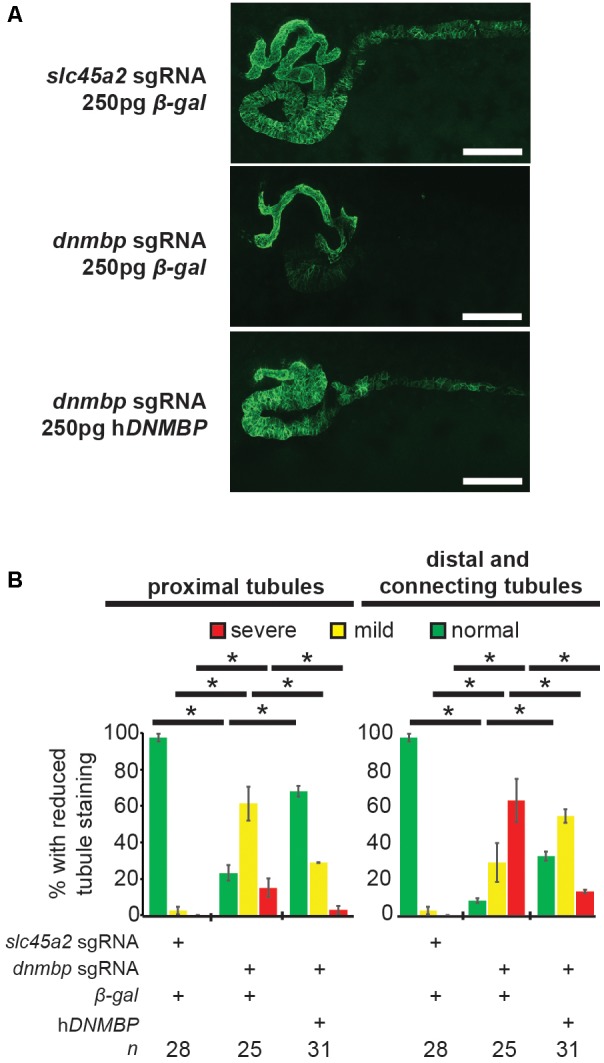
Human *DNMBP* mRNA rescues kidney defects caused by CRISPR *dnmbp* knockout in *Xenopus* embryos. **(A)** Representative embryos showing that co-injection of *β-galactosidase* RNA with a sgRNA against *dnmbp* does leads to kidney defects in comparison to control embryos injected with a sgRNA against *slc45a2* and *β-galactosidase* RNA. Co-injection of human *DNMBP* mRNA with a sgRNA against *dnmbp* rescues the knockdown phenotype. Stage 40 embryos stained with antibody 3G8 to label the proximal tubule and antibody 4A6 to label the distal and connecting tubules. White bar is 200 μm. **(B)** Quantitation of the rescue phenotype. *n* = number of embryos across 2 replications. Error bars represent Standard error. ^∗^Significantly different, *p* < 0.05.

*dnmbp* knockout specificity was tested by attempting to rescue the knockout phenotype through co-injection of *dnmbp* sgRNA and Cas9 protein with human *DNMBP* RNA. Control embryos injected with *slc45a2* sgRNA, Cas9 protein and β-*galactosidase* RNA exhibited normal kidney development at stage 40–41 (Figure 6A,B), while embryos injected with *dnmbp* sgRNA, Cas9 protein and β-*galactosidase* RNA had decreased proximal, distal and connecting tubule development. In contrast, the *dnmbp* rescue embryos injected with *dnmbp* sgRNA, Cas9 protein and human *DNMBP* RNA had an intermediate phenotype, with a significant reduction in the proximal, distal and connecting tubule defects (Figure 6A,B). This result indicates that human *DNMBP* RNA is able to rescue the kidney defects seen in *dnmbp* CRISPR knockout embryos and suggests that the observed kidney phenotype is specific to *dnmbp* knockout.

### *DNMBP* Overexpression Results in Altered Pronephric Tubulogenesis

**FIGURE 7:**
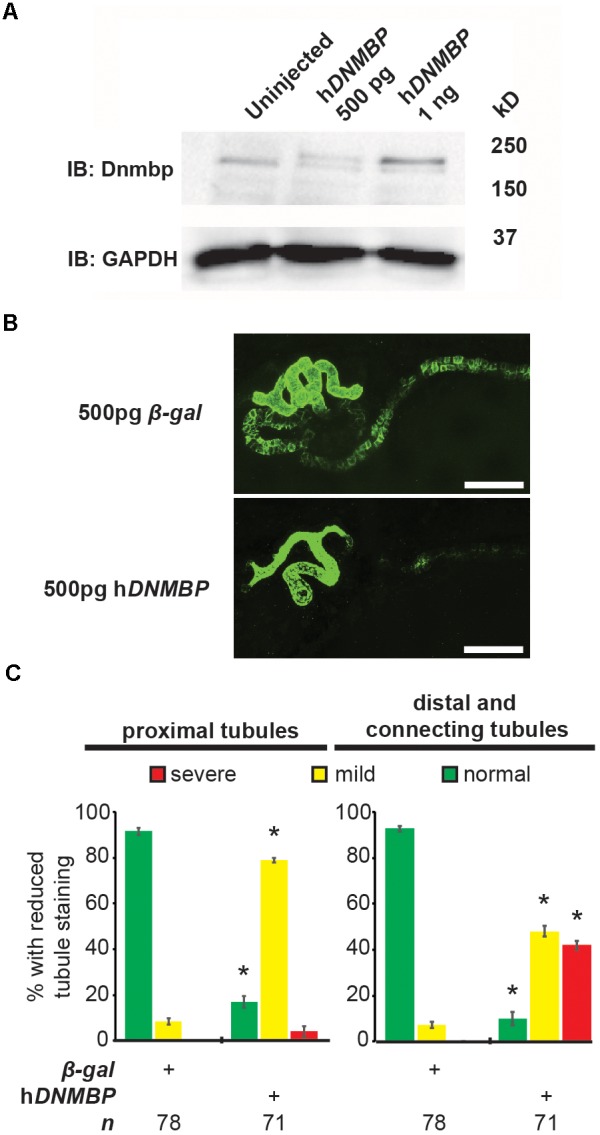
Overexpression of human *DNMBP* results in kidney tubule defects. **(A)** Western blot showing expression of both endogenous (*Xenopus*) Dnmbp (lower band) and exogenous (human) HA::DNMBP (upper band). Embryos injected at the one-cell stage and assayed at stage 10–12. One embryo equivalent loaded per lane. **(B)** Representative stage 40 embryos injected with either *β-galactosidase* control mRNA or human *DNMBP* mRNA. Overexpression of h*DNMBP* leads to disrupted kidney tubulogenesis in comparison to controls. Embryos injected at the 8-cell stage (blastomere V2) to target the kidney and stained with 3G8 antibodies to label the proximal tubule and 4A6 antibodies to label the distal and connecting tubules. White bar indicates 200 μm. **(C)** Quantitation of phenotypes showing that overexpression of h*DNMBP* leads to a reduction in proximal, distal and connecting tubule staining. *n* = number of embryos across 3 replications. Error bars represent Standard error. ^∗^Significantly different from control, *p* < 0.05.

Knockdown and knockout of Dnmbp leads to disrupted pronephric development, so studies were carried out to determine whether *DNMBP* overexpression also leads to tubulogenesis defects. Human *DNMBP* RNA was injected into single-cell embryos, which were then collected for protein lysate preparation at stage 10–12. DNMBP was detected by Western blot using an antibody against full-length human DNMBP protein that also recognizes *Xenopus* Dnmbp (Figure 7A). Note that the human DNMBP protein, which has an HA tag, runs at a slightly higher kD than the *Xenopus* Dnmbp protein. Therefore, both the human DNMBP band and the *Xenopus* Dnmbp band together represent the overall amount of Dnmbp in the embryo lysate of embryos overexpressing Dnmbp. Overexpression of human *DNMBP* RNA led to a decrease in endogenous embryo Dnmbp, with higher levels of human *DNMBP* overexpression leading to a greater decrease in endogenous Dnmbp protein. Overall, injection of human *DNMBP* RNA led to greater levels of Dnmbp protein in the resulting embryo lysates.

Similar to disruption of Dnmbp expression by knockdown or knockout, overexpression of Dnmbp led to kidney tubulogenesis defects (Figure 7B,C). Embryos were injected with either *β*-*gal* RNA as a negative control or human *DNMBP* RNA at the 8-cell stage (left V2 blastomere). Proximal tubule development was assessed using antibody 3G8, and distal and connecting tubule development were assessed using antibody 4A6 in stage 40–41 embryos. Dnmbp overexpression led to less convoluted proximal tubules with shorter branches in comparison to *β*-*gal* RNA control embryos. Likewise, Dnmbp overexpression led to less convoluted distal and connecting tubules, as well as decreased 4A6 staining indicating that the distal and connecting tubules were less differentiated than in β-*gal* RNA controls.

### Disruption of *dnmbp* Expression Does Not Alter Expression of Early Markers of Nephrogenesis

**FIGURE 8:**
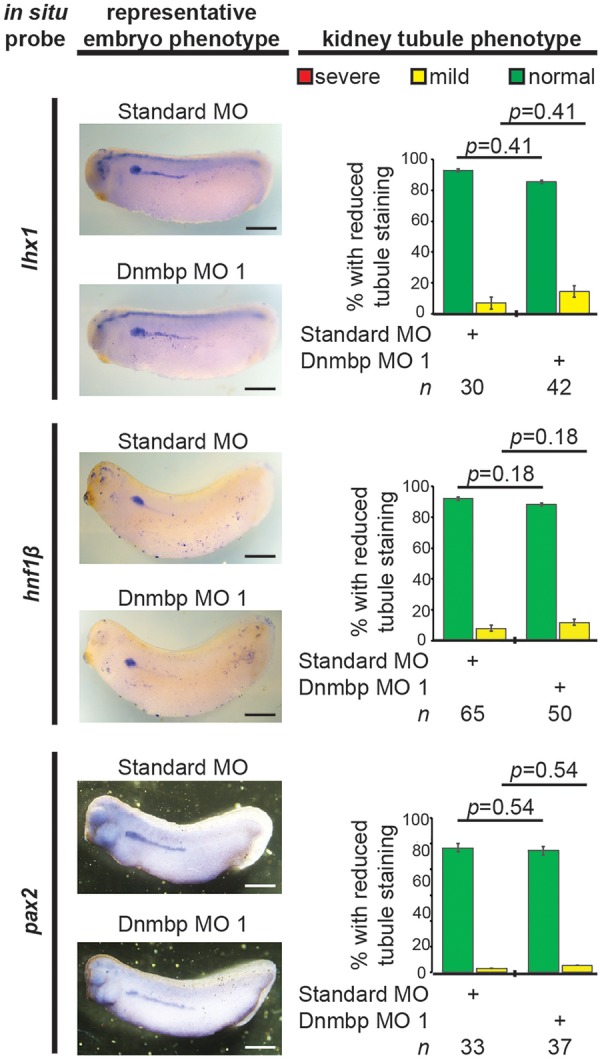
Dnmbp knockdown does not alter expression of early markers of kidney development, as assessed by *in situ* analysis. Embryos injected unilaterally at the 8-cell stage (blastomere V2) to target the kidney and reared to stage 29–30 for *lhx1* expression assessment, stage 32–33 for *hnf1β* expression assessment and stage 33–34 for *pax2* expression assessment. Representative embryo phenotypes shown. Scale bar indicates 500 μm. *n* = number of embryos across 2 (*pax2*) or 3 replications (*lhx1, hnf1β*). Error bars represent Standard error. ^∗^Significantly different from control, *p* < 0.05.

To further understand the role that Dnmbp plays in *Xenopus* kidney development, early markers of nephrogenesis were assessed by *in situ* hybridization. Embryos were injected with either Dnmbp MO 1 or Standard MO at the 8-cell stage (left V2 blastomere) and allowed to develop to stage 29–30 (*lhx1*), 32–33 (*hnf1β*), or 33–34 (*pax2*). Marker expression on the injected side was compared to the uninjected side of the embryo. Knockdown of these early markers of pronephric development (*lhx1, hnf1β*, and *pax2*) did not result in reduction of marker expression (Figure 8). This indicates that loss of *dnmbp* does not alter early kidney specification and development.

**FIGURE 9:**
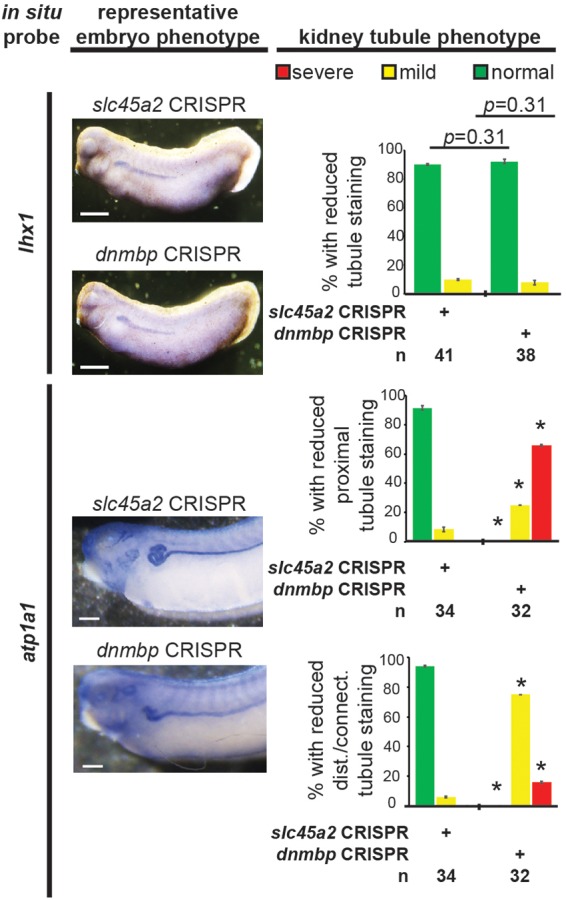
*dnmbp* CRISPR knockout does not alter expression of early markers of kidney development, however late marker expression is altered by *in situ* analysis. Embryos injected unilaterally at the 8-cell stage (blastomere V2) to target the kidney and reared to stage 29–30 for *lhx1* expression assessment and stage 40–41 for *atp1a1* expression assessment. Representative embryo phenotypes shown. Scale bar indicates 500 μm. *n* = number of embryos across 2 replications. Error bars represent Standard error. ^∗^Significantly different from control, *p* < 0.05.

To assess if the observed lack of early kidney defects in Dnmbp knockdown embryos also held true for *dnmbp* CRISPR knockout embryos, embryos were injected with a sgRNA against either *dnmbp* or *slc45a2* and Cas9 protein at the 8-cell stage. Embryos were assessed for *lhx1* staining by *in situ* hybridization at stage 29–30. Similar to Dnmbp knockdown embryos, there was no difference in *lhx1* staining between the *dnmbp* knockout embryos and the *slc45a2* knockout control embryos, suggesting that knockout of *dnmbp* gives a similar early kidney development phenotype to Dnmbp knockdown (Figure 9).

### Disruption of *dnmbp* Expression Perturbs Expression of Late Markers of Nephrogenesis

**FIGURE 10:**
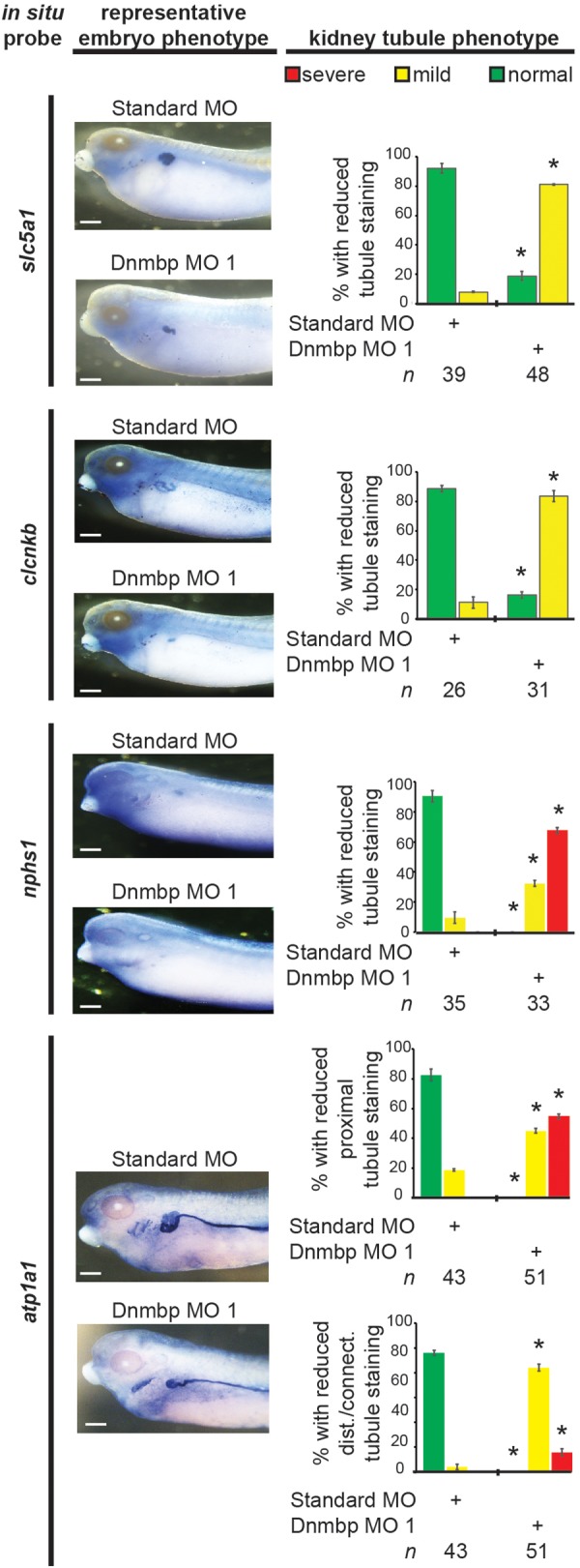
Dnmbp knockdown alters expression of late markers of kidney development, as assessed by *in situ* analysis. Embryos injected unilaterally at the 8-cell stage (blastomere V2) to target the kidney and reared to stage 40–41 for *slc5a1, clcnkb*, and *atp1a1* expression assessment or stage 36–37 for *nphs1* expression assessment. Representative embryo phenotypes shown. Scale bar indicates 500 μm. *n* = number of embryos across 2 (*slc5a1, clcnkb, nphs1*) or 3 replications (*atp1a1*). Error bars represent Standard error. ^∗^Significantly different from control, *p* < 0.05.

Next, late markers of nephrogenesis were assessed by *in situ* hybridization after injection of either Dnmbp MO 1 or Standard MO at the 8-cell stage (left V2 blastomere). The proximal tubule development of stage 40–41 embryos was assessed using an *in situ* probe against *slc5a1*. Although early stage embryos did not show nephrogenesis defects by *in situ* hybridization, Dnmbp knockdown embryos displayed a significant decrease in *slc5a1* staining on the injected side in comparison to the Standard MO control embryos at stage 40–41 (Figure 10). This phenotype corresponds to the phenotype seen by immunostaining stage 40–41 embryos with antibody 3G8, which also labels the proximal tubules (Figure 2C).

Distal and connecting tubule development in stage 40–41 embryos was assessed using a probe against *clcnkb*. Knockdown of Dnmbp resulted in decreased *clcnkb* staining of the distal and connecting tubules (Figure 10) in comparison to control embryos injected with Standard MO. The kidney tubules of these later stage Dnmbp knockdown embryos were less convoluted than the Standard MO control embryos, but did not exhibit a loss of staining as was observed using the 4A6 antibody (Figure 2C). This further indicates that the distal and connecting tubules are present in embryos depleted of Dnmbp, but that these tubules regions are less differentiated than they are in control embryos.

Glomus development was assessed using a probe against *nphs1*. Stage 36–37 Dnmbp knockdown embryos displayed a significant reduction in glomus development on the injected side of the embryo in comparison to control embryos injected with Standard MO (Figure 10). This indicates that Dnmbp is necessary for normal glomus development in addition to kidney tubulogenesis.

Finally, embryos were stained at stage 40–41 with *atp1a1*, which lightly stains the proximal tubules and strongly stains the distal and connecting tubules. Dnmbp knockdown embryos showed a marked decrease in proximal tubule expression of *atp1a1* (Figure 10). Similar to staining with *clcnkb*, the distal and intermediate tubules of Dnmbp knockdown embryos were less convoluted than Standard MO control embryo tubules and there was no distinct loss of distal and connecting tubule staining as was observed using the 4A6 antibody (Figure 2C). This result provides further evidence that the distal and connecting tubules of Dnmbp knockdown embryos are present but are less differentiated than control embryos.

Similarly, *atp1a1* staining was used to assess CRISPR knockout of *dnmbp* in stage 40–41 embryos. *dnmbp* knockout led to a decrease in proximal, distal and connecting tubule staining in comparison to *slc45a2* knockout controls (Figure 9). Like Dnmbp knockdown embryos, *dnmbp* knockout embryos displayed a reduction in the convolution of the distal and intermediate tubules.

### *dnmbp* Knockout Leads to Edema Formation

**FIGURE 11:**
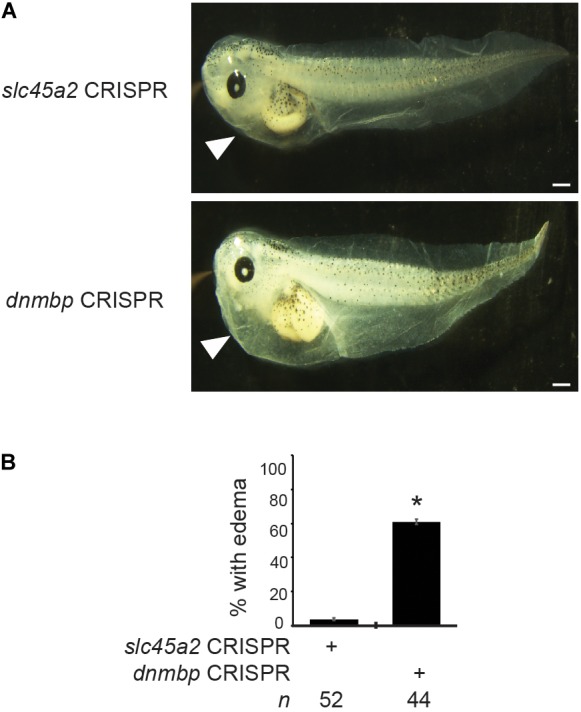
*dnmbp* CRISPR knockout results in edema formation. **(A)** Bilateral injection of *dnmbp* sgRNA and Cas9 protein into both ventral blastomeres at the 4-cell stage leads to edema formation in comparison to embryos injected with *slc45a2* sgRNA and Cas9 protein. Representative stage 45–46 embryos shown. White arrowhead indicates area of fluid accumulation. Scale bar indicates 500 μm. **(B)** Percent of embryos exhibiting edema formation. *n* = number of embryos across 3 replications. Error bars represent Standard error. ^∗^Significantly different from control, *p* < 0.05.

To determine if loss of Dnmbp leads to defects in kidney function, either *dnmbp* or *slc45a2* sgRNA and Cas9 protein were injected into both ventral blastomeres of 4-cell embryos. Embryos were allowed to develop to stage 45–46 and edema formation was assessed. Embryos were scored as positive for edema if fluid accumulation was present in the head and thorax (Corkins et al., 2018; DeLay et al., 2018b). A majority of *dnmbp* knockout embryos exhibited significant fluid accumulation in the thorax in comparison to control *slc45a2* knockout embryos (Figure 11).

## Discussion

Dnmbp was first discovered in a yeast two-hybrid screen designed to identify ligands that interact with EVL, a member of the Ena/VASP family of proteins (Salazar et al., 2003). Subsequent work determined that *DNMBP* transcripts were highly expressed in human kidney tissue, in addition to other organs such as the heart, brain, lungs and liver (Salazar et al., 2003). DNMBP directly interacts with actin regulatory proteins such as N-WASP and ENA/VASP and specifically activates CDC42, thereby playing a role in the assembly of actin (Salazar et al., 2003). In the kidney, Dnmbp depletion is associated with defects in ciliogenesis and tubulogenesis (Zuo et al., 2011; Baek et al., 2016).

Here, we describe the role that Dnmbp plays in *Xenopus* pronephric development. Dnmbp protein is present in whole embryo lysates starting in single cell embryos and continuing throughout kidney development. *In situ* hybridization showed that *dnmbp* transcripts are present in the developing kidney, as well as in head structures and somites. This finding is consistent with previous work showing that *DNMBP* transcripts are present in human kidney tissue and in the zebrafish pronephros, brain and eye (Salazar et al., 2003; Baek et al., 2016). The presence of Dnmbp during *Xenopus* embryonic development, and specifically in the kidney, suggests that it plays a role in kidney development.

Knockdown of Dnmbp with MOs shows that Dnmbp depletion leads to defects in *Xenopus* pronephric development. Proximal tubule branching was decreased upon *dnmbp* knockdown and the distal and connecting tubules were less convoluted than in control embryos. 3G8 and 4A6 antibodies were used to assess kidney development. Both of these antibodies label differentiated regions of the pronephric tubules, with antibody 3G8 staining the proximal tubules starting at stage 34 and 4A6 beginning to stain the distal and connecting tubules at stage 38, with complete staining by stage 41 (Vize et al., 1995). 4A6 antibody staining of the distal and connecting tubules was decreased in dnmbp knockdown embryos suggesting that these tubules were less differentiated than those of control embryos. These results are consistent with cell culture work that suggests that Dnmbp is necessary for tubulogenesis (Baek et al., 2016). Interestingly, previous work in zebrafish found no disruption of tubulogenesis upon *Dnmbp* MO knockdown (Baek et al., 2016). One possible explanation for this discrepancy is that the zebrafish pronephros has a less convoluted structure than the *Xenopus* pronephros (Drummond et al., 1998). Therefore, the decrease in tubule looping seen in *Xenopus* upon *dnmbp* depletion may not be apparent in the simpler zebrafish pronephros at the stages the authors examined. Dnmbp knockdown also resulted in glomus defects, similar to previous work that showed altered glomerulus development in zebrafish upon Dnmbp MO knockdown (Baek et al., 2016).

In addition to tubulogenesis and glomus defects, Dnmbp knockdown in *Xenopus* embryos results in altered primary cilia development within the kidney tubules. This result is similar to a previous report that MO knockdown of Dnmbp in zebrafish results not in loss of cilia, but in altered and disorganized primary cilia development within the pronephric tubules (Baek et al., 2016). Although primary cilia within the kidney tubule are altered by Dnmbp knockdown, there was no obvious defect in nephrostome development or in the multiciliated cells on the embryo epidermis (data not shown). Together, these data indicate that loss of Dnmbp does not result in the loss of cilia, but instead results in changes in primary cilia development within the developing kidney tubules.

Dnmbp loss also leads to functional defects of the kidney. To assess kidney function, edema development was assessed upon *dnmbp* knockout. The majority of the *dnmbp* knockout embryos displayed fluid accumulation in the thorax, indicative of kidney function defects. As edema formation may result from heart defects in addition to kidney defects, *dnmbp* knockout was targeted to the two ventral cells of 4-cell embryos. These cells will eventually give rise to the kidney, but do not contribute to heart formation (Moody, 1987a,b), thereby ruling out heart defects as a likely cause for edema formation. There are several possible explanations for the edema development observed due to *dnmbp* knockout. One possibility is that kidney tubule defects prevent normal fluid flow through the kidney tubules, resulting in edema. Another possibility is that although the kidney tubules are present, they may not be functional because they have not properly differentiated and epithelialized. Although cilia were present on the nephrostomes, it is possible that they have motility defects that prevent normal fluid flow through the kidney tubules. Finally, the glomus defects observed upon *dnmbp* knockout may also prevent proper fluid flow through the kidney, resulting in edema formation.

The specificity of the Dnmbp MOs was confirmed by rescue experiments, where human *DNMBP* RNA was able to rescue the knockdown phenotype. Additionally, CRISPR knockout of both *dnmbp* homeologs resulted in a similar phenotype to knockdown embryos and this phenotype could be rescued by co-injection with human *DNMBP* RNA. Together, these results suggest that disruption of Dnmbp expression leads to a decrease in pronephric tubulogenesis. Previous work suggests that defects in pronephric development can be secondary to defects in somite development (Mauch et al., 2000). To rule out the possibility that the kidney defects seen in knockdown embryos were the result of secondary defects due to somitogenesis defects, the somites of Dnmbp knockdown embryos were examined. There was no difference in somite development between Dnmbp knockdown and control embryos even though a co-injected tracer indicated that the somites were indeed subjected to Dnmbp knockdown, indicating that the kidney defects were not likely due to larger developmental defects. This point is especially important because *dnmbp* is expressed in the somites of developing *Xenopus* embryos.

Knockdown, knockout and overexpression of *dnmbp* led to similar defects in kidney development. All three of these manipulations led to a disruption in proximal tubule development, decreased looping of the distal and connecting tubules and less differentiation of the distal and connecting tubules. These results suggest that perturbations in the level of Dnmbp protein present during *Xenopus* embryonic developments result in pronephric defects.

Although Dnmbp knockdown results in pronephric tubule defects in stage 40 embryos, Dnmbp depletion did not result in a reduction of early markers of pronephric determination and patterning. This result indicates that the pronephros of Dnmbp knockdown embryos undergoes normal specification, and the pronephric defects seen in later embryos are due to changes in pronephric differentiation, ciliogenesis and/or function. The idea that the observed tubule defects are due to a lack of distal and connecting tubule differentiation is supported by our *in situ* hybridization results in stage 40 embryos, where probes for *clcnkb* and *atp1a1* completely stained the distal and connecting tubules of Dnmbp knockdown embryos, indicating that the distal and connecting tubules are indeed present. However, a loss of 4A6 staining of the distal and connecting tubules of stage 40 Dnmbp knockdown embryos indicates that the distal and connecting tubules are not completely differentiated. Therefore, in addition to the observed changes in primary cilia development within the pronephric tubules and decrease in kidney function as evidenced by edema formation, loss of Dnmbp results in a lack of pronephric tubule differentiation. In conclusion, we demonstrate for the first time that Dnmbp is essential for normal vertebrate tetrapod kidney development and function. Our findings suggest that depletion of Dnmbp does not affect pronephric specification, but instead alters differentiation, ciliogenesis and function of the developing kidney tubules.

## Author Contributions

BD performed microinjections, *in situ* hybridization, Western blots, designed *dnmbp* sgRNA and *in situ* probes, conducted sequencing and TIDE analysis, and wrote the manuscript. TB performed initial overexpression Western blot and phenotypic experiments. RM conceived of the project, designed Dnmbp MOs and oversaw the experiments, and manuscript preparation. All authors edited the article and approved of the final version.

## Conflict of Interest Statement

The authors declare that the research was conducted in the absence of any commercial or financial relationships that could be construed as a potential conflict of interest.

## Supplementary Material

FIGURE S1 *In situ* hybridization of both homeologs of *dnmbp*. Antisense probes (AS) labeling *dnmbp* expression in the pronephros, head structures and somites. Sense (S) probes shown as a control for non-specific probe binding were processed in parallel with the AS probes. Scale bar indicates 500 μm.

FIGURE S2 Knockdown of Dnmbp results in reduced kidney tubulogenesis. **(A)** Unilateral injection of 20 ng Dnmbp MO 2 into blastomere V2 at the 8-cell stage leads to defects in kidney tubulogenesis in comparison to embryos injected with Standard MO. Antibody 3G8 used to label the lumen of the proximal tubule, antibody 4A6 used to label the distal and connecting tubules. memRFP used as an injection tracer. White bar indicates 200 μm. ^∗^Indicates injected side of embryo. **(B)** Knockdown of Dnmbp leads to reduced expression of differentiated kidney tubule markers in comparison to embryos injected with Standard MO. *n* = number of embryos across 3 replications. Error bars represent Standard error. ^∗^Significantly different from control, *p* < 0.05.

FIGURE S3 Primers used to amplify regions of *dnmbp* for TIDE analysis. **(A)** DNA sequence of the region surrounding exon 3 of *dnmbp.L* indicating sgRNA and sequencing primer binding sites. **(B)** DNA sequence of the region surrounding exon 3 of *dnmbp.S* indicating sgRNA and sequencing primer binding sites.
