# Supplementary figures and images for: Dynamin Binding Protein Is Required for Xenopus laevis Kidney Development
Source: Front Physiol. 2019 Feb 26;10:143. doi: 10.3389/fphys.2019.00143 (PMC6399408; doi:10.3389/fphys.2019.00143)

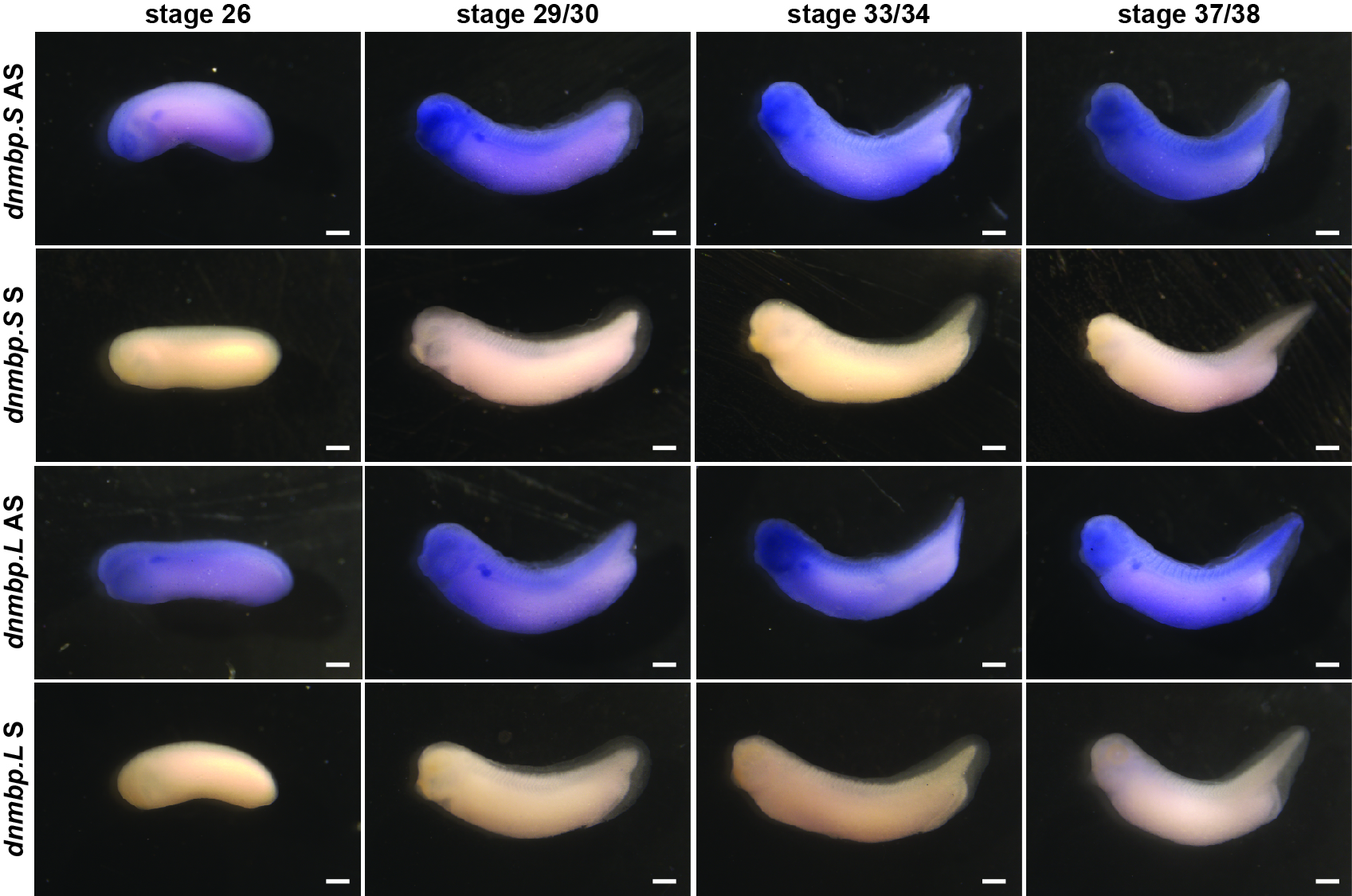

Supplement: FIGURE S1 — In situ hybridization of both homeologs of dnmbp. Antisense probes (AS) labeling dnmbp expression in the pronephros, head structures and somites. Sense (S) probes shown as a control for non-specific probe binding were processed in parallel with the AS probes. Scale bar indicates 500 μm. [file Image_1.JPEG]

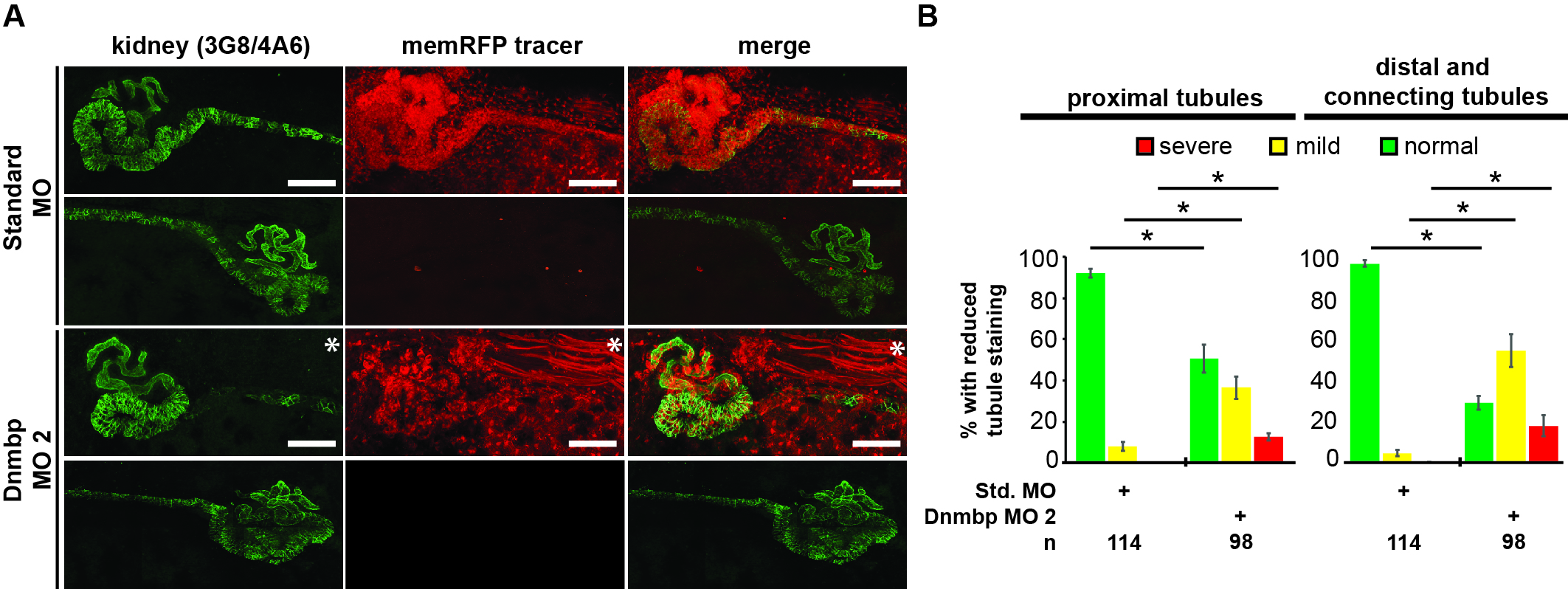

Supplement: FIGURE S2 — Knockdown of Dnmbp results in reduced kidney tubulogenesis. (A) Unilateral injection of 20 ng Dnmbp MO 2 into blastomere V2 at the 8-cell stage leads to defects in kidney tubulogenesis in comparison to embryos injected with Standard MO. Antibody 3G8 used to label the lumen of the proximal tubule, antibody 4A6 used to label the distal and connecting tubules. memRFP used as an injection tracer. White bar indicates 200 μm. ∗Indicates injected side of embryo. (B) Knockdown of Dnmbp leads to reduced expression of differentiated kidney tubule markers in comparison to embryos injected with Standard MO. n = number of embryos across 3 replications. Error bars represent Standard error. ∗Significantly different from control, p < 0.05. [file Image_2.JPEG]

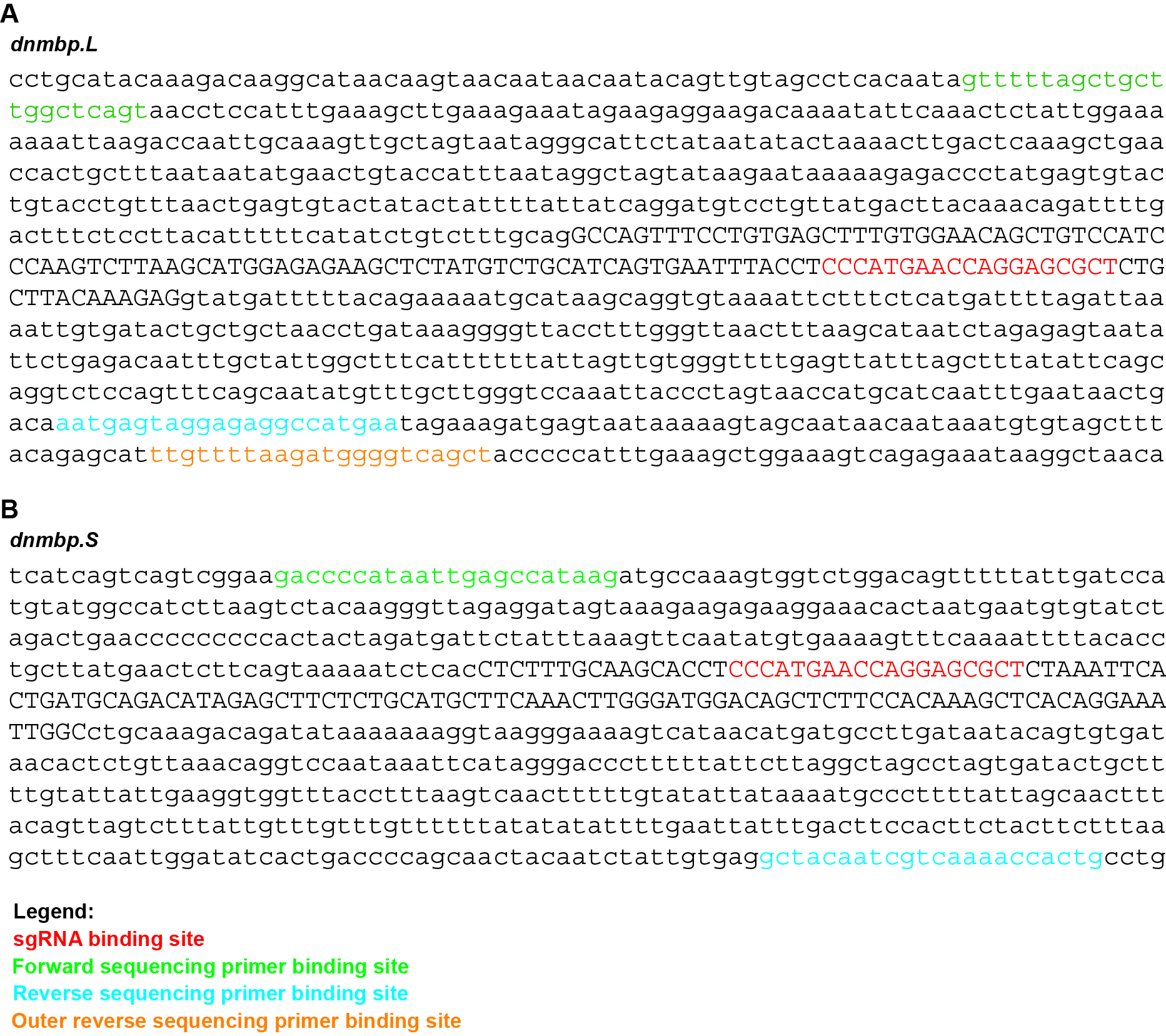

Supplement: FIGURE S3 — Primers used to amplify regions of dnmbp for TIDE analysis. (A) DNA sequence of the region surrounding exon 3 of dnmbp.L indicating sgRNA and sequencing primer binding sites. (B) DNA sequence of the region surrounding exon 3 of dnmbp.S indicating sgRNA and sequencing primer binding sites. [file Image_3.JPEG]
